# Oral Health Status of Children Living in Gorom-Gorom, Oudalan District, Burkina Faso

**DOI:** 10.1155/2010/597251

**Published:** 2010-06-09

**Authors:** Clelia Mazza, Laura Strohmenger, Guglielmo Campus, Maria Grazia Cagetti, Filippo Caruso, Poul Erik Petersen

**Affiliations:** ^1^Department of Odontontostomatology, Orthodontics and Surgery, Second University of Naples, Naples, Italy; ^2^WHO Collaborating Centre for Epidemiology and Community Dentistry, Milan, Italy; ^3^Dental Institute, University of Sassari, Sassari, Italy; ^4^Global Oral Health Programme, Department of Chronic Disease and Health Promotion, World Health Organization, Geneva, Switzerland

## Abstract

*Aim*. In order to set up the needs for intervention and to plan oral health prevention and care programmes, this paper aims to describe the oral health status and behaviour in children living in the municipality of Gorom-Gorom, Burkina Faso. *Design*. The sample size was 692 children, 334 females (48.3%) and 358 males (51.7%). Clinical and oral health related behaviours were collected. *Results*. 83.4% of the children were caries-free. Fluorosis was recorded in 41.3% of the sample, while only 37.9% of children showed healthy gingival condition. Toothbrushing was reported by 35.7% of children. A statistically significant association was found between caries experience and cleanliness of hands. Community Periodontal Index was statistically associated to toothbrushing and chewingstick use. *Conclusion*. As suggested by WHO's global strategies, integrated primary oral health care and services meeting the dental needs of the local population are necessary for children living in this area of Africa.

## 1. Introduction

Nowadays, the African region still faces the wors health conditions in comparison with the rest of the world. Thirty-two African countries are among the world's forty-eight least developed nations [1] while literature reports that the 90% of the eleven million of children (up to 5 years age) dying every year for neonatal disorders, diarrhea, acute respiratory infections and malaria are found in the poorest counties worldwide [2]. Poverty, underdevelopment and un-nourishment together with wars, drought, lack of health and educational services are some of the more relevant health determinants in the region. Since common risk factors, such as social, economic, and political ones, are worldwide recognized both for some of the most common noncommunicable diseases and oral diseases [3], awareness is generally rising about the need to integrate general health action plans with oral health interventions [4].

Dental caries and periodontal disease prevalence seems not to be so severe in Africa as in the rest of the world while oral disease pattern is not homogeneous across the region and need to be assessed in each community [5].

In 2006, Thorpe highlighted the importance of having reliable data about the oral health profile of different communities across Africa as a prerequisite to develop and improve effective intervention programmes to address oral health needs [5]. The major challenges for the near future will be to turn knowledge and experience of disease prevention into action. Oral health advances and knowledge have not been achieved yet in developing countries. Clear disparities widened the gap between poor and rich countries. In May 2007, the WHO General Assembly (WHA60.17 resolution) claimed for considering mechanism to provide coverage of the population with essential oral health care to promote the availability of oral health services that should be directed towards diseases prevention and health promotion for poor and disadvantage, countries. For these countries it is needed to consider, especially for schoolchildren, the development and implementation of preventive programmes, as a part of activities in health promoting schools, aiming to introduce healthy lifestyle and self-care practices in children [6, 7]. 

Burkina Faso, previously known as Upper Volta, is a low-income country in West Africa having 50% of the population living with less then 1 US$ per day, characterized by poor sanitary conditions and lack of services [8]. In Burkina Faso, as well as in other African countries, a national oral health system is developing, and health promotion and oral disease prevention are the main goals health authorities are promoting. In order to plan community oral health promotion programmes, it is necessary to assess the oral health situation of the target population. Although the DMFT is reported to be rather low (0.7 at 12 years old and 1.9 at 18 years), periodontal diseases are extensively diffused and oral manifestation of AIDS, gangrenous infections (ANUG) and NOMA still represents a serious concern [4, 9].

The Oudalan province is one of the 45 provinces of Burkina Faso, located in the northern part of Burkina Faso, and it is included within Sahel, the semi-arid belt running across sub-Saharan Africa. Oudalan is divided into 5 departments: Deou, Gorom-Gorom, Markoye, Oursi and Tin-Akof. Almost 400 kilometers far from Ouagadougou, Burkina Faso capital city, there is the municipality of Gorom Gorom, a small town surrounded by tens of villages and gatherings in the savannah. No current oral health services are available both for town and villages inhabitants and the first available dental clinic is 60 kilometres faraway. Consequently in Oudalan province, the population has a poor or lack access to dental care. People suffer with tooth pain, often with no opportunity for relief, without making a long and costly journey. Dental visits are mainly performed for symptomatic reasons and not for restorative reasons.

In order to set up the needs for intervention and to plan oral health prevention and care programmes, this paper aims to describe the epidemiological oral health status and behaviour in children living in the municipality of Gorom-Gorom, Oudalan province, Sahel region, Burkina Faso.

## 2. Material and Methods

### 2.1. Study Area

This survey included the most relevant subgroups of the population (always 12 years age included) according to the World Health Organization [10].

**Figure 1:**
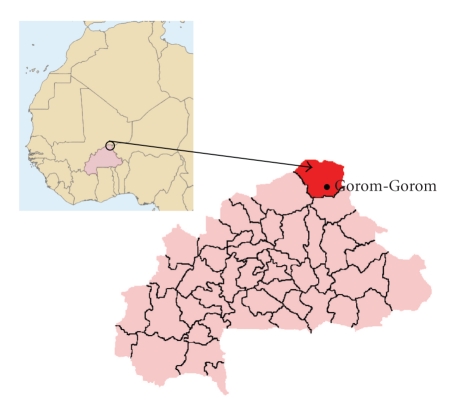
Burkina Faso, Oudolan Province.

The survey was carried out as a cross-sectional study, during two months, in 2008 in children living in the municipality of Gorom Gorom, Oudalan province, Sahel region, Burkina Faso (Figure 1). The Municipality counts about 80,000 inhabitants living in 82 villages and in Gorom Gorom capital [11]. Children came from three main different ethnic groups. No data about the fluoride concentration in the area were available. Three samples of well water were collected near schools location. The fluoride concentration was carried out using an Orion model 96.09 fluoride ion selective electrode and an Orion model 900200 double junction plastic body Ag/Cl reference electrode with Orion model 290 mV digital meter. Fluoride content was quite high, with a mean value of 1.7 ± 0.6 ppm. No direct previous contact had been with a dental worker before for the children population, but traditional healer serves for people oral care needs.

### 2.2. Selection of the Sample

Children aged 5-6 and 12 years from 11 schools and 9 villages of the Municipality were included in the survey. The 5-6 year group is relevant for the examination of deciduous teeth while the 12 year for the permanent ones [10]. Moreover, the 12-year group is relevant for international data comparison.

An authorization for the survey was obtained both from national health, educational authorities and local administrators. During a school meeting, parents or guardians were informed about the aim of the survey, describing the dental visit procedure and explaining the questionnaire items; after all, their verbal agreement was obtained. All children's parents or guardians consented to clinical examination and oral interview. In schools the collection was conducted in the presence of teachers and in rural villages in the presence of the local chief. 

The sample size was 692 children; 334 females (48.3%) and 358 males (51.7%). The subjects were then categorized for age into two groups: 338 subjects aged 5-6 years (48.8%) and 354 subjects aged 12 years (51.2%).

### 2.3. Methods

Children were included in the two age groups using the local educational authorities records. When it was not possible, due to the lack of data collection (i.e. remote villages and gatherings), children with a deciduous full-arch were considered 5 years old, those with first permanent molars not yet fully erupted (under occlusal edge) 6 years old, those with second permanent molars not completely yet erupted 12 years old.

One examiner assisted by a local interpreter carried out all clinical assessments. the month before the beginning of the study, the examiner took part to a calibration session organized in the WHO Collaboration Centre of Milan. The examiner received training and intra-examiner reliability was assessed. Forty subjects (20 for each age group) were re-examined after 72 hours by the examiner. Intraexaminer reproducibility was assessed as percent agreement and Cohen's Kappa statistics. The percent agreement was high (Cohen's Kappa 0.84). 

The visits were performed on a simple bed or school table using a plain mirror, a WHO-CPI probe, paper hand towels, and gauzes to remove debris from around the teeth. The instruments were first washed in a conventional manner with soap and then sterilized with a solution of paracetic acid (Parasafe). 

An artificial head-light source was used. The examiner was seating beside the head of the child. The examination setting was always arranged according to the local condition and avoiding natural light sources and crowding.

Data were collected using a modified WHO examination form [10]. The interpreter was previously trained on how to fill the epidemiologic form. General information about the patient like age, gender, ethnic group, and living conditions was collected. 

The recorded clinical conditions were dental caries experience (dmft/DMFT), gingival condition (presence of plaque and/or calculus), and fluorosis following WHO indication [10]. Dental caries was registered when decay at the dentinal lesion level was found [12]; gingival conditions were scored into three classes following the Community Periodontal Index: score 0 (healthy), score 1 (gingival bleeding at probing), and score 2 (calculus). Fluorosis was assessed by the Dean's Index.

Cleanliness of the hands was recorded as follows: score 1 (clean), score 2 (dirty) and score 3 (crusted) as a proxy of hygiene condition of the subject.

Oral health related behaviours were collected by a questionnaire directed to children and parents or guardians. It was divided into different items: oral hygiene habits (tooth-brushing habit, use of a toothbrush or a chewing stick, use of fluoridated toothpaste) and dietary habits (number of meal *per* diem).

### 2.4. Statistical Analysis

Both descriptive and analytic approaches were used for the data analysis. Caries distribution according to age was calculated as mean ± standard errors (SE). Caries prevalence was calculated as the number of subjects with dt > 0 and DMFT > 0 compared to the whole sample. Contingency tables by age groups were performed by contingence table and *χ* ^2^ test. Odds Ratio (OR) was calculated between caries prevalence and other variables (age, gender, fluorosis, and cleanliness of hands) as well as for periodontal condition (with gender, caries experience, cleanliness of hands, and oral hygiene habit). 

All the analyses were carried out using Stata SE software 8.2. A *P*-value less than .05 was considered statistically significant.

## 3. Results

Overall, 83.4% of the children showed no signs of obvious caries. No score for missing or filled tooth was assigned, so data reported only caries activity (DT) prevalence. In the two age groups (5-6 and 12 years), dt (mean ± SE) was 1.5 ± 0.1 and 0.1 ± 0.3, while DT (mean ± SE) was 0.2 ± 0.3 and 0.5 ± 0.6. Table 1 describes the entire caries experience by age, gender, fluorosis, and cleanliness of hands as count (percent) and odds ratio (OR) for each category. 

According to Dean's Index, 61 children (8.8%) showed no sign of fluorosis or it was uncertain; for 343 children (49.7%) it was very mild, for 213 (30.9%) it was mild, for 72 (10.4%) it was moderate or severe (Table 2). 

Overall, 37.9% (150 of 5-6 years and 112 of 12 years) of children showed healthy gingival condition. Plaque was scored in 27.8% of cases (107 of 5-6 years and 85 of 12 years), while calculus in 34.4% of cases (81 of 5-6 years and 157 of 12 years). 

Children reported having 3 meals *per* day in 97.1% of the sample (672) and 2 meals *per* day in 2.9% (20 children).

Children were reported to brush their teeth in 35.7% (247) of the sample: 7.1% (49 children) with a toothbrush and 28.6% (198 children) with a chewing stick. Only 6.1% (42) use fluoridated toothpaste.

Table 3 shows an association between Community Periodontal Index scores and toothbrushing habit and chewing stick usage, respectively. No association was found between CPI scores and gender, caries experience and cleanliness of hands, respectively (*P* > .05) (*data not in table*). 

Significant trends in proportion (*P* < .05) of Community Periodontal Index, across categories of exposure, were found for these two variables.

## 4. Discussion

Oral health status responds to many determinants of disease. Nowadays, the usual etiologic approach to tooth decay or periodontal disease is revised considering social, cultural, environmental determinants as well as oral health systems orientation. Many more other studies have been carried out on oral health determinants and today oral pathology is considered a social exclusion pointer [13].

Oral health is today an unfulfilled need for most people worldwide especially for those belonging to the most disadvantaged groups of society both in low and high income countries. The burden of oral disease is growing due to a lack of proper community oral health programmes. The evidence for a link between sociobehavioural risk factors and dental decay was pointed out, underlining a social gradient in caries prevalence [14, 15].

This model highlights three distal determinants for oral health: health systems and oral health services, sociocultural risk factors and environmental risk factors. 

The aim of this study was to provide an overview of the oral health condition of children in the region of Burkina Faso in order to assist an intervention of oral health promotion.

Few epidemiological data are available for oral health condition in Burkina Faso as a whole; the latest ones date back to the 90s [16] and reported general poor oral health hygiene, significant prevalence of periodontal diseases, and generally low prevalence of dental decays (with higher levels in urban areas). No specific data for the Oudalan Province was found at the moment of this survey nor conducted and no previous contact has had been with a dental worker for the children population. This situation points out the urgent need to implement integrated primary oral health care and oral health services that meet the needs of the local population as suggested by WHO's global strategies [17].

The main finding of this study was the low caries prevalence (more than three quarters of the children were without obvious caries “caries free”) probably linked to the quite high concentration of fluoride in well water. 

Similarly, the high fluoride concentration is the cause of the high level of fluorosis observed. The absence of filled or missing for caries scored teeth (M/F score) highlights the absence of accessible services even for urgent treatments.

Comparing with other data collected in southwest Burkina Faso in 1999 [16], scoring caries prevalence at 38% of 6 year-aged children, this study reports a very lower rate. 

The majority of children resulted to have 3 meals *per* day: diet is not varied, poor in quantity, and low in calories. The most common food is a milk soup, millet, and sugar, badly affecting their growth. Cariogenic snacks are not available except for a minority of children living in Gorom Gorom, while it is a common habit to drink sweet herb brew. Only a few children from Gorom Gorom reported to use a fluoridated toothpaste due to a lack of availability as well as for general high cost, as reported in literature [18, 19]. Only one third of children were found to brush their teeth. The use of a toothbrush is quite common in town, while it is almost absent in villages. A common habit of the population is to use a chewing stick from acacia tree; a statistically significant association has been found between periodontal condition and chewing habit. 

No clear significance was found between periodontal condition and cleanliness of hands, while it was found for caries experience. Less than fifty percent of children have clean hands considering that drought and lack of water are common. Moreover, this survey was conducted during the dry season when highly severe diseases as acute and chronic intestinal infections spread and put life of children at serious risk.

The present study maybe the typical limitation of a cross-sectional survey, like the low number of participants with respect to total population. Cross-sectional survey displays only “static” data. However, in this report the rate of participants was high and this is the first report about children oral condition living in this area. 

From a public health point of view this study supports recommendation from the most authoritative international organizations claiming the urgent need to change policies and strategies for oral health.

In May 2007, the Assembly of the World Health Organisation declared that “the economic burden of oral diseases is expected to rise rapidly globally, above all, among disadvantaged and poor population groups, at least until programmes for the prevention of oral disease are promoted”.

Moreover, as reported by The Lancet in 2009, “training more dentists and building dental clinics—the western curative model of care—is costly and unrealistic in most low-income and middle-income countries” [20].

Primary Health Care Approach (basic and sustainable care, community participation, appropriate technology, maternal and childhood health focus and prevention integrated with educational and social sectors) is the only realistic one to address oral health needs for communities at any latitude of the world. 

 A good first step to improve the oral condition of the Oudalan children should be that local authorities would accept and organize a dental service based on Basic Package Of Oral Care (BPOC) developed by the WHO Collaborating Centre of Nijmegen [21].

Although it is recognised that communities and the circumstances in which African populations live are widely different, to win the many challenges ahead for Africa, it is important to build up strategies to provide equitable and universal access to oral health care services and to promote preventive oral health programs in order to significantly reduce the impact of oral diseases in African populations.

## Figures and Tables

**Table:** (a)

	dmft/DMFT > 0	dmft/DMFT = 0	OR	CI_95%_	*P*-value
	*n* (%)	*n* (%)			
Age					
5-6-year-old *n* = 338	34 (10.1)	304 (89.9)	0.1	0.1-0.2	<.05
12-year-old *n* = 354	81 (22.9)	273 (77.1)	0.3	0.2–0.4	

**Table:** (b)

	dmft/DMFT > 0	dmft/DMFT = 0	OR	CI_95%_	*P*-value
	*n* (%)	*n* (%)			
Gender					
Male	64 (17.9)	294 (82.1)	0.2	0.2-0.3	>.05
Female	51 (15.3)	283 (84.7)	0.2	0.1-0.2	

**Table:** (c)

Fluorosis	dmft/DMFT > 0	dmft/DMFT = 0	OR	CI_95%_	*P*-value
	*n* (%)	*n* (%)			
No sign or Uncertain	11 (18.0)	50 (82.0)	0.2	0.1–0.4	<.05
Very slight	44 (12.8)	299 (87.2)	0.2	0.1-0.2	
Slight	40 (18.8)	173 (81.2)	0.2	0.2-0.3	
Moderate or Severe	19 (26.4)	53 (73.6)	0.4	0.2–0.6	

**Table:** (d)

Cleanliness of hands	dmft/DMFT > 0	dmft/DMFT=0	OR	CI_95%_	*P*-value
	*n* (%)	*n* (%)			
Clean	35 (11.9)	258 (88.1)	0.1	0.1-0.2	<.05
Dirty	43 (21.7)	155 (78.3)	0.3	0.2–0.4	
Crusted	37 (18.5)	163 (81.5)	0.3	0.1–0.3	

**Table 2:** Fluorosis levels in the two-age sample (percent in colon).

Fluorosis (Dean Index)	5-6-year-old	12-year-old
	*n*° (%)	*n*° (%)
No sign or Uncertain (0)	28 (45.9)	33 (54.1)
Very mild (1)	201 (58.6)	142 (41.4)
Mild (2)	82 (38.5)	131 (61.5)
Moderate or Severe (>2)	24 (33.3)	48 (66.7)

No responders 3 (0.4%).

*χ* ^2^ = 29.3 (3) *P* = .01.

**Table:** (a)

CPI	Toothbrushing YES	Tooth-brushing NOT	OR	CI_95%_	*P*-value
0	91 (13.1%)	171 (24.7%)	0.5	0.4–0.7	<.05
1	53 (7.7%)	139 (20.1%)	0.4	0.3–0.5	
2	103 (14.9%)	135 (19.5%)	0.8	0.6–1.0	

**Table:** (b)

CPI	Chewing stick YES	Chewing-stick NOT	OR	CI_95%_	*P*-value
0	66 (9.5%)	196 (28.3%)	0.3	0.3–0.4	<.05
1	44 (6.4%)	148 (21.4%)	0.3	0.2–0.4	
2	88 (12.7%)	150 (21.7%)	0.6	0.5–0.8	
